# Activation of the methylation cycle in cells reprogrammed into a stem cell-like state

**DOI:** 10.18632/oncoscience.280

**Published:** 2016-01-05

**Authors:** Salvador Fernández-Arroyo, Elisabet Cuyàs, Joaquim Bosch-Barrera, Tomás Alarcón, Jorge Joven, Javier A. Menendez

**Affiliations:** ^1^ Unitat de Recerca Biomèdica (URB-CRB), Institut d’Investigació Sanitaria Pere i Virgili (IISPV), Universitat Rovira i Virgili, Reus, Spain; ^2^ Campus of International Excellence Southern Catalonia, Tarragona, Spain; ^3^ ProCURE (Program Against Cancer Therapeutic Resistance), Metabolism & Cancer Group, Catalan Institute of Oncology (ICO), Girona, Spain; ^4^ Molecular Oncology Group, Girona Biomedical Research Institute (IDIBGI), Girona, Spain; ^5^ Department of Medical Oncology, Catalan Institute of Oncology (ICO), Girona, Spain; ^6^ Department of Medical Sciences, Medical School, University of Girona, Girona, Spain; ^7^ Computational and Mathematical Biology Research Group, Centre de Recerca Matemàtica (CRM), Barcelona, Spain

**Keywords:** iPS cells, stem cells, one-carbon metabolism, S-adenosylhomocysteine, Homocysteine

## Abstract

Generation of induced pluripotent stem (iPS) cells and cancer biogenesis share similar metabolic switches. Most studies have focused on how the establishment of a cancer-like glycolytic phenotype is necessary for the optimal routing of somatic cells for achieving stemness. However, relatively little effort has been dedicated towards elucidating how one-carbon (1C) metabolism is retuned during acquisition of stem cell identity. Here we used ultra-high pressure liquid chromatography coupled to an electrospray ionization source and a triple-quadrupole mass spectrometer [UHPLC-ESI-QqQ-MS/MS] to quantitatively examine the methionine/folate bi-cyclic 1C metabolome during nuclear reprogramming of somatic cells into iPS cells. iPS cells optimize the synthesis of the universal methyl donor S-adenosylmethionine (SAM), apparently augment the ability of the redox balance regulator NADPH in SAM biosynthesis, and greatly increase their methylation potential by triggering a high SAM:S-adenosylhomocysteine (SAH) ratio. Activation of the methylation cycle in iPS cells efficiently prevents the elevation of homocysteine (Hcy), which could alter global DNA methylation and induce mitochondrial toxicity, oxidative stress and inflammation. In this regard, the methyl donor choline is also strikingly accumulated in iPS cells, suggesting perhaps an overactive intersection of the de novo synthesis of choline with the methionine-Hcy cycle. Activation of methylogenesis and maintenance of an optimal SAM:Hcy ratio might represent an essential function of 1C metabolism to provide a labile pool of methyl groups and NADPH-dependent redox products required for successfully establishing and maintaining an embryonic-like DNA methylation imprint in stem cell states.

## INTRODUCTION

Cellular metabolism is emerging as an essential driving force for the nuclear reprogramming of somatic cells into pluripotent embryonic stem cell-like states [1–7]. The accumulating state of evidence also strengthens the close similarity of the metabolic switches required for generation of induced pluripotent stem (iPS) cells and cancer biogenesis. Accordingly, cellular reprogramming might a provide a useful platform to investigate the specialized metabolic programs responsible for the acquisition of cancer stem cell (CSC)-like states endowed with self-renewal and tumor/metastasis-initiating potential.

Although mechanistic interpretations cannot yet clearly establish which facets of metabolic retuning are necessary during reprogramming and which are consequences rather than causal factors for stemness, studies on the metabolome of iPS cells have focused predominantly on molecular mechanisms of mitochondria-related glucose utilization, including glycolysis, oxidative phosphorylation and reactive oxygen species (ROS) generation [8–13]. In contrast, there has been surprisingly little attention given to the study of one-carbon (1C) metabolism, a bi-cyclic cellular pathway involving the folate and methionine cycle that generates the universal methyl donor molecule S-adenosylmethionine (SAM). Accordingly, a major function of 1C metabolism is to supply the methyl groups required for a number of biological reactions, including DNA and histone methylation. Given its importance for epigenetic regulation of cell fate [14–16], we hypothesized that 1C metabolism plays a central role during reprogramming. We here applied targeted metabolomics using ultra-high pressure liquid chromatography coupled to an electrospray ionization source and a triple-quadrupole mass spectrometer [UHPLC-ESI-QqQ-MS/MS] to examine how the folate/methionine bi-cyclic 1C metabolome becomes altered during reprogramming of somatic cells into iPS cells.

## RESULTS

### iPS cells exhibit an increased index of SAM/SAH-driven cellular methylation potential

**Figure 1:**
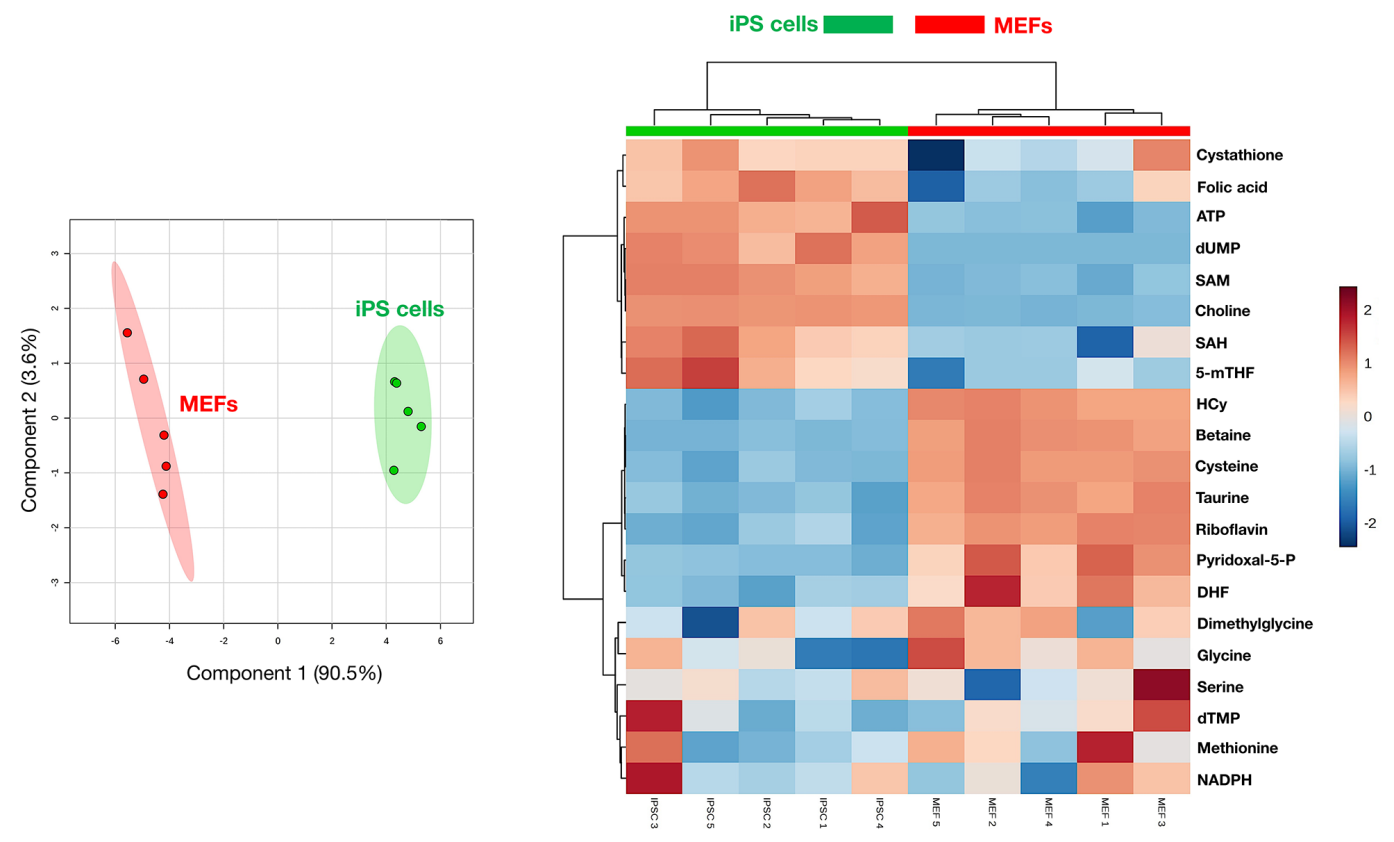
1C metabolomic profiling of iPS cells 2D (A) and 3D (B) PLS-DA models for the parental MEFs and iPS cells groups. C. Heatmap visualization and hierarchical clustering analysis with MetaboAnalyst's data annotation tool. Rows: metabolites; Columns: samples; color key indicates metabolite expression value (i.e., blue: lowest; red: highest).

An UHPLC-MS based metabolomic approach was carried out to differentiate 1C metabolite alterations in iPS cells from parental mouse embryonic fibroblasts (MEFs). Sample clustering patterns by using the partial least squares-discriminant analysis (PLS-DA) method showed a clear separation between the iPS group and the original MEFs group when using two-dimensional (Figure 1, left panel) score plots. Indeed, MetaboAnalyst's data annotation tools (http://www.metaboanalyst.ca) revealed differences between the two groups; thus, Heatmap visualization, a commonly used approach for unsupervised clustering, showed distinct segregation of metabolites for the iPS and parental MEFs (Figure 1, right panel).

**Figure 2:**
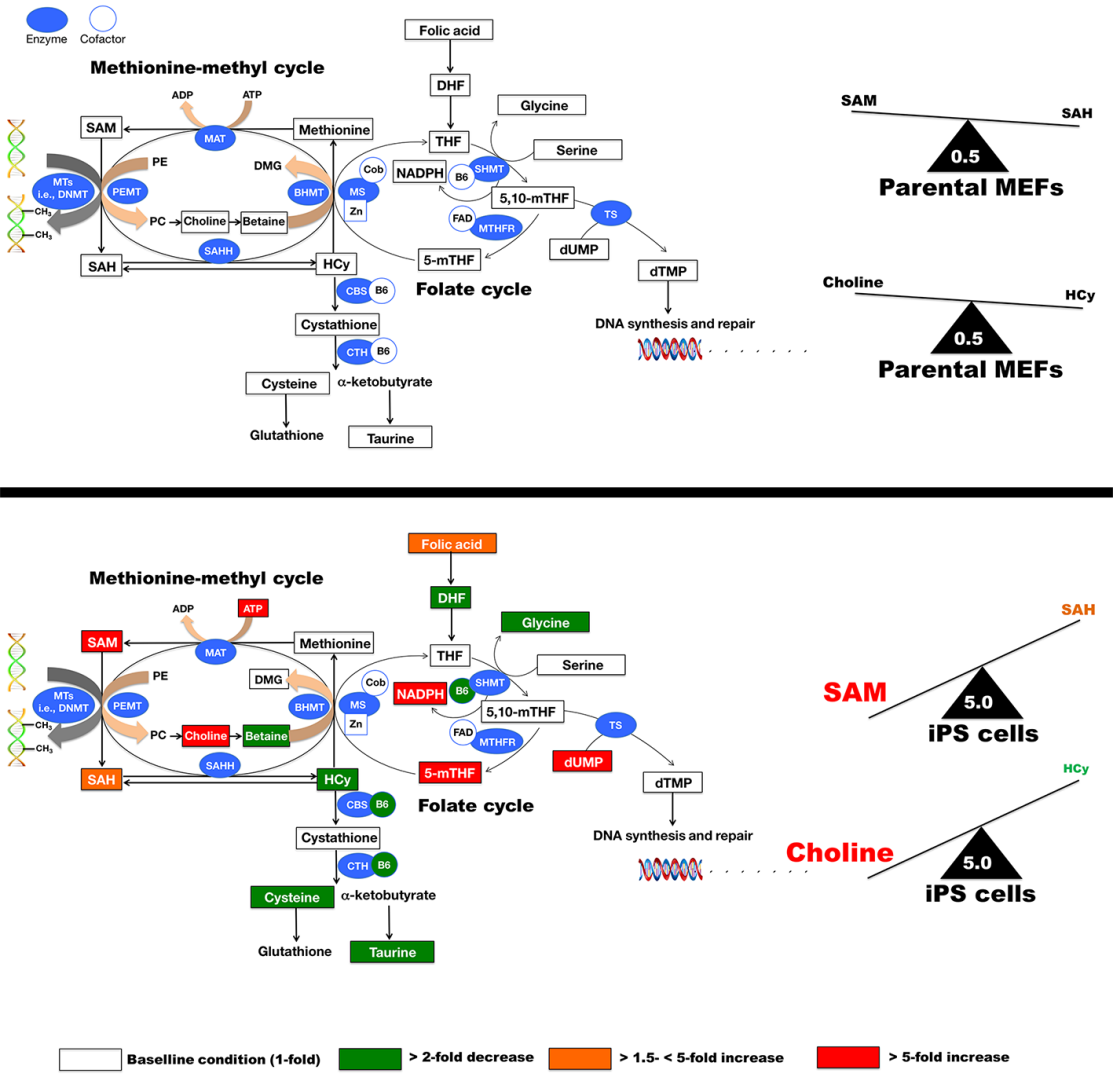
Metabolomic profile of the methionine/folate bi-cyclic 1C metabolism in iPS cells SAM serves as the methyl donor for numerous methylation reactions, including DNA methylation and synthesis of phosphatidylcholine. During methyl group donation, SAM is converted into SAH, a potent competitive inhibitor of many methyltransferases including DNMTs. Demethylated SAM then is further processed to Hcy, which in turn is converted back to methionine either through a folate-dependent mechanism (the folate cycle on the right side of the schematic, in which 5-mTHF is the methyl donor in the MS pathway) or a folate-independent mechanism (in the left side of the schematic, in which betaine is the methyl donor in the BHMT pathway). MAT: Methionine adenosyltransferase; PEMT: Phosphatidylethanolamine-N-methyl transferase; MTs: Methyl transferases; DNMTs: DNA methyl transferases; SAHH: SAH hydrolase; Hcy: Homocysteine; BHMT: Betaine homocysteine methyltransferases; MS: Methionine synthase; SHMT: serine hydroxymethyltransferase; MTHFR: methylenetetrahydrofolate reductase; 5-mTHF: 5-methyltetrahydrofolate; 5,10-mTHF: 5,10-methylenetetrahydrofolate; Cob: Cobalamine; Zn: Zinc; CBS: Cystathione-β-synthase; DMG: Dimethylglycine; FAD: Flavin adenine dinucleotide; PE: Phosphatidylethanolamine; PC: Phosphatidylcholine; TS: Thymidylate synthase.

Compared with parental fibroblasts, we found that the abundance of certain metabolites belonging to the methionine-methyl cyclic (also called methionine remethylation) pathway of 1C metabolism were changed significantly in iPS cells, as illustrated by a major accumulation (approx. 14-fold increase) of SAM, the major methyl donor for DNA methylation and synthesis of phosphatidylcholine (PC) (Table 1, Figure 2). Levels of S-adenosylhomocysteine (SAH), the product of these transmethylation reactions, were also significantly elevated in iPS cells compared with parental fibroblasts (approx. 1.6-fold increase) but to a notably lesser extent, suggesting active conversion of SAM to SAH during methylation of DNA and other macromolecules in iPS cells. Indeed, nuclear reprogramming was accompanied by a marked increase in the “methylation capacity” measured as the SAM:SAH ratio, which was 10-fold higher in iPS cells (5.0) than in parental fibroblasts (0.5) (Figure 2).

**Table 1:** Concentration and fold change of 1C metabolites in parental MEFs and their iPS cell derivatives

Metabolite	[Metabolite]^a^ MEFs	[Metabolite]^a^ iPS cells	Fold-change [iPS]/[MEFs]
5-Adenosyl-methionine (SAM)	0.30 ± 0.04	4.2 ± 1.1	13.8
NADPH	0.05 ± 0.03	0.43 ± 0.06	7.6
Choline	2454 ± 109	15391 ± 600	6.3
5-methyl-tetrahydrofolate (5-mTHF)	0.006. ± 0.001	0.021 ± 0.008	3.5
ATP	2.5 ± 0.2	7.3 ± 1.6	2.8
5-Adenosyl-homocysteine (SAH)	0.6 ± 0.1	1.0 ± 0.1	1.6
Folic acid	0.19 ± 0.05	0.27 ± 0.02	1.5
dUMP	N.D.	8.2 ± 2.7	>>
Cystathionine	0.3 ± 0.1	0.38 ± 0.03	1.25
Serine	37 ± 5	36.3 ± 1.4	1.0
Thymidine 5-phosphate (dTMP)	0.64 ± 0.09	0.61 ± 0.09	1.0
Dimethylglycine	108 ± 5	102 ± 16	0.9
Methionine	2.5 ± 0.5	2.2 ± 0.5	0.9
Glycine	42 ± 19	23 ± 5	0.6
Pyridoxal 5-phosphate (B6)	2.2 ± 0.5	0.86 ± 0.05	0.4
Betaine	1.9 ± 0.1	0.52 ± 0.02	0.3
Homocysteine (HCy)	0.49 ± 0.04	0.13 ± 0.02	0.3
Riboflavin (B2)	0.037 ± 0.001	0.006 ± 0.002	0.2
Taurine	24 ± 3	3.7 ± 0.7	0.15
Cysteine	0.32 ± 0.02	0.052 ± 0.008	0.1
Dihydrofolate (DHF)	0.039 ± 0.01	0.008 ± 0.003	0.06
Cyanocobalamin (B12)	N.D.	N.D.	-
Folinic acid (5-formyl-tetrahydrofolate)	N.D.	N.D.	-
Methylcobalamine	N.D.	N.D.	-
Tetrahydrofolate (THF)	N.D.	N.D.	-

a [Metabolite] in mean (μM/mg of protein) ± SD. N.D. Not detectable. Decimals were set according to the first significant digit of the measure SD.

### iPS cells reduce the levels of homocysteine

SAH is reversibly hydrolyzed to adenosine and homocysteine (Hcy), which not only plays a critical role in the regulation of methylation but also sustains the flux of methionine sulfur toward cysteine. Indeed, Hcy is metabolized through two major pathways: methylation and transsulfuration. Under normal physiological conditions, adenosine and Hcy are rapidly removed, driving the reaction toward SAH hydrolysis; however, this reaction can be reversed upon the pathological accumulation of Hcy, leading to SAH accumulation. Accordingly, the maintenance of correct methylation status critically depends not only on the SAM:SAH ratio, but also on the levels of Hcy [17–19]. We observed that iPS cells exhibited a strong reduction in their intracellular Hcy levels, by approx. 3.7-fold compared with parental fibroblasts (Table 1, Figure 2). Because the concentration of Hcy was significantly diminished and the transsulfuration flow, as measured by the concentration of cystathione and its derivatives (e.g., cysteine, taurine), was not activated but rather reduced upon nuclear reprogramming, one could speculate that recycling of Hcy back to methionine and SAM was overactive in iPS cells. Indeed, iPS cells accumulated higher levels of the methyl provider 5-methyl-tetrahydrofolic acid (5-mTHF) (3.5-fold increase; Table 1, Figure 2), which might suggest that an active coupling of the folate cycle -wherein the synthesis of 5-mTHF is catalyzed by methylenetetrahydrofolate reductase (MTHFR) from 5,10-mTHF-to the methionine cycle -wherein 5-mTHF donates a carbon through methylation of Hcy and generates methionine in a process catalyzed by the 5-methyltetrahydrofolate-Hcy methyltransferase (MTR or methionine synthase [MS])-might occur during somatic cell reprogramming. Independent experiments using iPS cells from different fibroblast source confirmed that a greater than *10*-*fold increase* in the levels of the methyl donor 5-mTHF can be observed in some iPS clones when compared to the parental fibroblast cells (data not shown). Nevertheless, iPS cells appears to be highly dependent on certain NADPH-regenerating folate pathway enzymes and other metabolic pathways that contribute to the synthesis and regeneration of NADPH, which exhibited a strong accumulation (approx. 8-fold increase) in iPS cells relative to parental fibroblasts (Table 1).

### iPS cells accumulate the methyl donor choline

In our hands, 5-mTHF was the sole significantly altered metabolite in the folate cyclic pathway of 1C metabolism, suggesting an asymmetric. Therefore, we speculated that the preferential usage of methionine metabolism and the methionine re-methylation pathway may account for the syntehsis and regeneration of SAM in iPS cells. Although the MTHFR-driven, folate-dependent pathway is usually considered the major route for remethylation of Hcy to methionine, it can also be derived through a folate-independent pathway involving betaine as the methyl donor (Figure 2). In this alternative pathway, the enzyme betaine-Hcy S-methyltransferase (BHMT) transfers methyl groups from betaine (trimethylglycine) to Hcy to regenerate methionine with dimethylglycine as the other product [20–22]. Betaine is synthesized from choline by the enzymes choline oxidase and betaine aldehyde dehydrogenase. Further metabolism of dimethylglycine generates two 1C units, thereby recovering all 3 methyl groups donated from SAM to form choline. Thus, the metabolism of choline intersects with the methionine-Hcy cycle at the de novo synthesis of choline, in which the sequential transfer of methyl groups from SAM to phosphatidylethanolamine by phosphatidylethanolamine-N-methyl transferase (PEMT) leads to the de novo synthesis of choline in phosphatidylcholine (PC), and through the betaine-dependent remethylation of Hcy. Because betaine is a methyl donor for Hcy remethylation via BHMT, choline, the precursor of betaine, should be negatively related to Hcy concentrations [23, 24]. Interestingly, our targeted metabolomics approach revealed that iPS cells accumulated considerably larger amounts of choline (approx. 6.2-fold increase), but notably decreased their endogenous pool of betaine (approx. 3.9-fold decrease) compared with parental fibroblasts (Table 1, Figure 2). Indeed, the nuclear reprogramming process was accompanied by a marked increase in the choline:Hcy ratio, which was 10-fold higher in iPS cells (5.0) than in parental fibroblasts (0.5) (Figure 2).

## DISCUSSION

**Figure 3:**
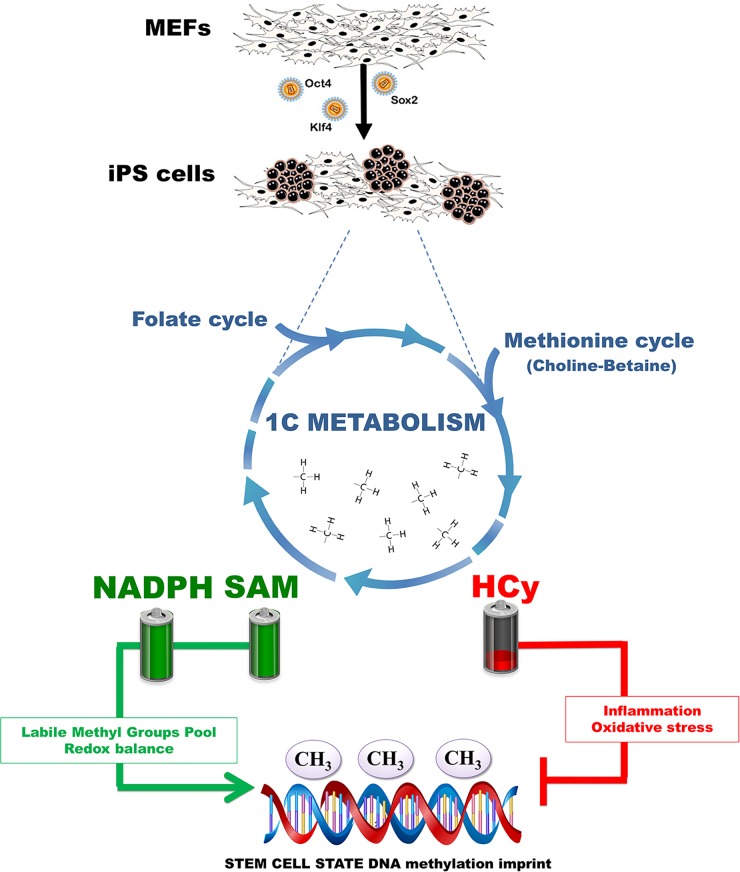
1C metabolism in iPS cells: Balancing methylogenesis, redox, and inflammation Activation of 1C metabolism and the process of methylogenesis during nuclear reprogramming of somatic cells into iPS cells might ensure the correct correlation between the SAM/Hcy cycle and the DNA methylation imprint of stem cell states by providing a labile pool of methyl groups and NADPH-dependent redox products while avoiding the pro-oxidant and pro-inflammatory effects of Hcy.

We have demonstrated that the methylation cycle is activated in iPS cells. Because SAM is the universal substrate for enzymatic methylation all transmethylation reactions in cells and the SAM/SAH ratio is important for regulating methylation since methyltransferases are product-inhibited by SAH, our findings reveal that iPS cells optimize the synthesis of SAM to maintain a high SAM/SAH ratio. However, as SAM is not the final product in the cell and could be further converted to SAH and other metabolites, it is biochemically difficult to accumulate SAM to high levels as it occurs in iPS cells. Moreover, as one molecule of NADPH is consumed for each turn of the folate cycle to reduce THF, the maintenance of the high NADPH levels in iPS cells should demand the activation of efficient regeneration steps. Because the availability of NADPH provides redox carriers for biosynthetic reactions, the active flow of carbon atoms through 1C metabolism might establish a possible mechanistic link not only with epigenetic state but also with the redox status of iPS cells (Figure 3).

Because the rate-limiting step for synthesis of glutathione (GSH) is the pool size of cellular cysteine, it might be tempting to suggest that iPS cells bypass the requirement for GSH to counteract oxidative stress when considering their low levels of cysteine. However, it should be noted that embryonic stem cells and iPS cells express low levels of the cystine transporter (xCT) and cannot maintain cellular cysteine and GSH pools [25]. Not surprisingly, fibroblasts have been used as feeder cells for iPS cells based on their ability to stably supply cysteine. Although our feeder-free culture conditions of iPS cells contained 2-mercaptoethanol, which can improve the redox balance by enhancing cysteine uptake to maintain cellular GSH levels, a constitutively low iPS cells’ cystine transport activity similar to that of mouse lymphoid cells can explain the lowered cysteine levels in the pluripotent phenotype [25]. Forthcoming studies should elucidate whether availability of GSH precursors such as cystine and/or redox cycling from glutathione disulfide (GGSG) alters the de novo synthesis and/or the salvage replenishing of GSH during nuclear reprogramming of somatic cells into iPS cells.

iPS cells efficiently prevent the occurrence of elevated Hcy levels, which could alter global DNA methylation and induce mitochondrial toxicity, oxidative stress and inflammation. Indeed, increased Hcy levels are known to associate with decreased SAM and increased SAH levels, and SAH-related DNA hypomethylation is a major biochemical mechanism underlying the well-established epigenetic role of hyperhomocysteinemia in atherogenesis and other disorders [26–29]. In mammals, Hcy can be re-methylated via two pathways. In most somatic cells, a methyl group derived from serine is carried by folic acid as 5-mTHF and transferred to Hcy. An alternative pathway utilizes betaine, whereby a methyl group is donated to Hcy by the enzyme BHMT. The folate-independent, betaine-dependent pathway of SAM synthesis has generally been considered to be restricted to liver and possibly also kidney. Although the relative importance of the folate-and betaine-dependent pathways for SAM generation remains to be investigated, our findings reveal for the first time that the intersection of the de novo synthesis of the methyl donor choline with the methionine-Hcy cycle appears to be overactivated in iPS cells. The observed increase of endogenous choline and decrease in betaine might be consistent with the rapid metabolism of betaine by BHMT at the iPS stage, suggesting a stem-cell phenotype-related activation of liver-specific BHMT. Interestingly, two studies have revealed that BHMT is unexpectedly active in the mouse blastocyst and promotes the development of the inner cell mass [30, 31], where ES cells reside. Additionally, gene expression profiling in mouse ES cells highlighted the activation of Bhmt1 and Bhmt2 genes [32], both encoding for BHMT.

Because methionine adenosyltransferase, an enzyme involved in amino acid biosynthesis, has been shown to actively convert methionine and ATP into SAM for DNA methyltransferases (DNMTs) and other transmethylation reactions [33], our findings strongly suggest that iPS cells appear to have redundant regulatory systems to maintain constant high levels of SAM, a key regulator for maintaining survival and self-renewal of undifferentiated pluripotent stem cells, and low levels of Hcy, whose accumulation may elicit aberrant DNA methylation patterns (Figure 3). Further studies should elucidate whether iPS cells are unusual in being able to restrict Hcy accumulation while generating high levels of SAM by asymmetrically feeding the methionine-methyl cycle preferentially using methionine metabolism coupled to the choline-betaine axis of methyl group donors, rather than the ubiquitous folate-dependent mechanism. This process would contribute not only to SAM generation, but also to the necessary synthesis of purines and DNA synthesis and repair enzymes in highly proliferative iPS cells [34, 35].

We acknowledge that a major limitation of the steady-state targeted metabolomics employed in this study is that we are unable to unambiguously discern whether the observed changes in metabolites are associated with alterations in production or utilization. In this framework, metabolic flux experiments utilizing 13C-labeled carbon tracers could, however, elucidate the extent to which the folate cycle and the betaine/BHMT pathway differentially contribute to a labile pool of methyl groups required for establishing an embryonic-like DNA methylation imprint in induced stem cell states. Because it is increasingly recognized that iPS cells-related metabolic features can be commonly found in so-called cancer stem cells (CSCs) and viceversa, forthcoming studies should decipher whether the recently discovered ability of mitochondrial 1C metabolism to increase the capacity of metastasizing cancer cells to withstand oxidative stress [36–38] might reflect an essential function of 1C metabolism to provide a labile pool of methyl groups and NADPH-dependent redox products required for successfully establishing and maintaining stem cell states including those of CSCs.

We are currently embarking on one of the cancer field's biggest challenges, namely the understanding of how cellular metabolism or certain classes of metabolites can directly influence chromatin structure and the functioning of epi-transcriptional circuits to causally drive tumor formation [39–41]. The metabolic state is now considered a hallmark of pluripotency [42] and the interplay between cellular metabolism and the core epigenetic codes, DNA methylation and histone modification, can be better viewed as an integrated metaboloepigenetic dimension of CSCs, which we have recently termed cancer metabostemness [43–45]. As tumorigenesis shares possible common features and mechanisms with iPS and dysregulated metabolism including one-carbon metabolism [46, 47] has the power to control both genetic and epigenetic events in cells [48], our study showing activation of the methylation cycle in cells reprogrammed into a stem cell-like state suggests that the homeostatic relationship between one-carbon metabolism and genome methylation might offer renewed opportunities to therapeutically target CSCs.

## MATERIALS AND METHODS

### Reagents

The following chemicals were purchased from Sigma-Aldrich (St. Louis, MO): methanol and acetonitrile (MS grade), ammonium acetate, ascorbic acid, β-mercaptoethanol, formic acid and standards, 5-adenosyl-methionine, 5-adenosyl-homocysteine, ATP, betaine, choline, cyanocobalamin, cystathionine, cysteine, dihydrofolate, dimethylglycine, dUMP, folic acid, folinic acid, glycine, homocysteine, methionine, methylcobalamine, methyl-tetrahydrofolate, NADPH, pyridoxal 5-phosphate, riboflavin, serine, taurine, tetrahydrofolate, thymidine 5-phosphate and Dulbecco's modified Eagle's medium mixed 1:1 with Ham's F-12 (DMEM/F12). Ultrapure type 1 water was obtained from a Milli Q water system (Merck Millipore, Darmstadt, Germany). Matrigel (growth factor-reduced) was purchased from BD Biosciences (San Jose, CA). Recombinant basic fibroblast growth factor (bFGF) was purchased from Mylteni (San Diego, USA).

### Generation of iPS cells

Mouse primary iPS cell were created generated by transducing mouse embryonic fibroblasts (MEFs) with murine retroviruses that individually encode the transcription factors Oct3/4, Sox2, and Klf4 following a previously described protocol [49, 50]. Characterization of bona fide iPSC cells colonies was carried out by analyzing pluripotent marker expression by alkaline phosphatase (AP) staining using the StemTAG™ Alkaline Phosphatase Staining and Activity Assay Kit (Cell Biolabs, Inc., San Diego, CA; Cat. No. CBA-302) and the expression of Oct3/4, Nanog, Sox2, and Ssea-1 by immunocytochemistry. To generate feeder-free iPS cells cultures for metabolomic analysis, culture plates were coated with 0.3 mg/mL Matrigel (growth factor-reduced, BD Biosciences, San Jose, CA) at room temperature in the hood for 1–3 hours. Unbound Matrigel was aspirated, and the cells were washed with DMEM/F12 medium. iPS cells were seeded on Matrigel-coated plates and cultured in complete KSR medium composed by DMEM/F12 (high glucose) supplemented with knock-out serum replacement (KRS, 15%), LIF 1000 U/mL, non-essential amino acids, glutamax, beta-mercaptoethanol, and penicillin/streptomycin. Medium replacement was performed every 2 days.

### Metabolomic analysis

Metabolite extraction was carried out by resuspending the cell pellets in 200 μL of methanol/water (8/2) containing 1% ascorbic acid and 0.5% β-mercaptoethanol. Cells were lysed by three cycles of freezing-thawing using liquid N2 and sonicated with three cycles of 30 seconds. Samples were maintained on ice for 1 minute between each sonication step. Subsequently, proteins were precipitated over 2 hours at −20°C, centrifuged at 14000 rpm for 10 minutes at 4°C and the supernatant transferred into a vial. For the quantification of metabolites involved in 1-C metabolism, samples (2 μL) were injected into an Agilent 1290 infinity ultra-high pressure liquid chromatograph (UHPLC) coupled with an iFunnel electrospray ionization source (ESI) to a 6490 triple quadrupole mass spectrometer (QqQ-MS) (Agilent Technologies, Santa Clara, CA). The UHPLC was equipped with a binary pump (G4220A), an autosampler (G4226A) termostatized at 4°C and an Acquity UPLC HSS T3 C18 column (2.1 mm x 100 mm; 1.8 μm) (Waters Corporation, Milford, MA). The mobile phase consisted of A: 50 mM ammonium acetate + 0.2% formic acid and B: acetonitrile, at a flow rate of 0.2 mL/min. The gradient used was: 0 min, 0% B; 1 min, 10% B; 6 min, 25% B; 8 min, 65% B; 9 min, 100% B, 11 min, 100% B; 12 min, 0% B. A postrun of 2 minutes in initial conditions was used for column conditioning. For the ESI source, the optimized parameters were: gas temperature 290°C; gas flow 18 L/min; nebulizer 20 psi; sheath gas temperature 350°C; sheath gas flow 10 L/min. For the QqQ-MS, running in positive mode, the parameters were set as follow: capillary at 3500 V; nozzle voltage at 750 V; High pressure radiofrequency at 150 V. The scan was carried out in multiple reaction monitoring (MRM) mode. For each metabolite, the precursor and product ions, collision energy and retention time are summarized in Table S1 (Supplementary material).

### Data analysis

Raw data were processed and compounds were detected and quantified using the Qualitative and Quantitative Analysis B.06.00 software (Agilent Technologies), respectively. MetaboAnalyst 3.0 (http://www.metaboanalyst.ca/) was used to generate scores/loading plots and Heatmaps [51].

## SUPPLEMENTARY MATERIAL TABLE


