# Supplementary material for: Activation of the methylation cycle in cells reprogrammed into a stem cell-like state
Source: Oncoscience. 2016 Jan 5;2(12):958–67. doi: 10.18632/oncoscience.280 (PMC4735514; doi:10.18632/oncoscience.280)
Supplement: Supplementary file 1 [file oncoscience-02-958-s001.pdf]

Activation of the methylation cycle in cells reprogrammed into a stem cell-like state

Supplementary Material

Table S1. Retention time, precursor ion, product ion and collision energy established for each metabolite analyzed

5-adenosyl-homocysteine (SAH)	3.83	385	136	30
5-Adenosyl-methionine (SAM)	3.72	399	250	20
5-methyl-tetrahydrofolate (5-mTHF)	4.01	460	313	30
ATP	3.74	508	136	30
Betaine	1.90	118	58	30
Choline	1.31	104	60	30
Cyanocobalamin (B12)	4.59	1355	1209	70
Cystathionine	1.70	223	88	30
Cysteine	1.37	122	59	30
Dihydrofolate (DHF)	3.97	444	178	30
Dimethylglycine	1.83	104	58	30
dUMP	3.60	309	81	20
Folic acid	3.71	442	295	15
Folinic acid (5-formyl-tetrahydrofolate)	3.93	474	327	20
Homocysteine (HCy)	2.04	136	56	20
Glycine	1.25	76	30	18
Methionine	3.03	150	56	20
Methylcobalamine	5.75	1345	1184	65
NADPH	3.76	745	136	30
Pyridoxal 5-phosphate (B6)	2.01	248	150	20
Riboflavin (B2)	5.40	377	243	30
Serine	1.74	106	60	15
Taurine	1.80	126	65	40
Tetrahydrofolate (THF)	3.96	446	99	40
Thymidine 5-phosphate (dTMP)	3.74	323	81	25
